# Conformable AlN Piezoelectric Sensors as a Non-invasive Approach for Swallowing Disorder Assessment

**DOI:** 10.1021/acssensors.0c02339

**Published:** 2021-05-19

**Authors:** Lara Natta, Francesco Guido, Luciana Algieri, Vincenzo M. Mastronardi, Francesco Rizzi, Elisa Scarpa, Antonio Qualtieri, Maria T. Todaro, Vincenzo Sallustio, Massimo De Vittorio

**Affiliations:** † Istituto Italiano di Tecnologia, Center for Biomolecular Nanotechnologies, Arnesano 73010, Italy; ‡ Piezoskin S.r.l., Lecce 73100, Italy; § Consiglio Nazionale delle Ricerche, c/o Campus Ecotekne, Istituto di Nanotecnologia Via Monteroni, Lecce 73100, Italy; ∥ Hospital Unit Phoniatrics and Communication Disorders, Rehabilitation Department, ASL Lecce, Lecce 73100, Italy; ⊥ Università del Salento, Lecce 73100, Italy

**Keywords:** piezoelectric sensor, aluminum nitride, flexible electronics, deglutition analysis, laryngeal movement

## Abstract

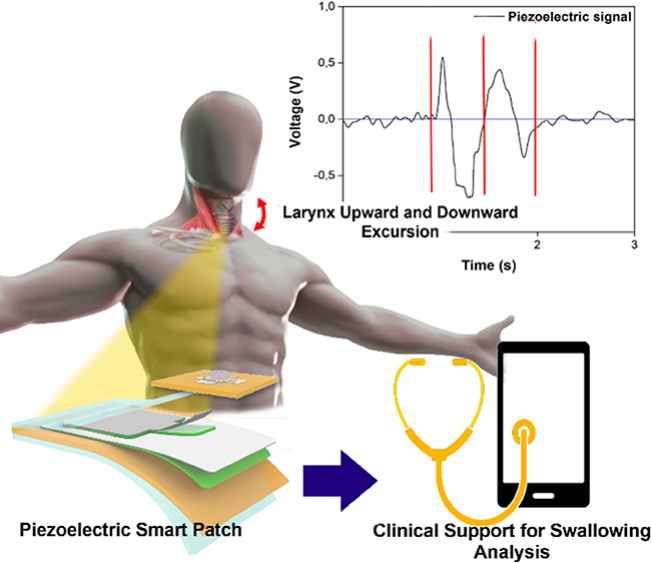

Deglutition disorders (dysphagia) are common symptoms of a large number of diseases and can lead to severe deterioration of the patient’s quality of life. The clinical evaluation of this problem involves an invasive screening, whose results are subjective and do not provide a precise and quantitative assessment. To overcome these issues, alternative possibilities based on wearable technologies have been proposed. We explore the use of ultrathin, compliant, and flexible piezoelectric patches that are able to convert the laryngeal movement into a well-defined electrical signal, with extremely low anatomical obstruction and high strain resolution. The sensor is based on an aluminum nitride thin film, grown on a soft Kapton substrate, integrated with an electrical charge amplifier and low-power, wireless connection to a smartphone. An ad-hoc designed laryngeal motion simulator (LMS), which is able to mimic the motions of the laryngeal prominence, was used to evaluate its performances. The physiological deglutition waveforms were then extrapolated on a healthy volunteer and compared with the sEMG (surface electromyography) of the submental muscles. Finally, different tests were conducted to assess the ability of the sensor to provide clinically relevant information. The reliability of these features permits an unbiased evaluation of the swallowing ability, paving the way to the creation of a system that is able to provide a point-of-care automatic, unobtrusive, and real-time extrapolation of the patient’s swallowing quality even during normal behavior.

Deglutition is the complex series of processes allowing the transportation of food from the oral cavity to the stomach.^1,2^ Via a wave-like elevation, the tongue pushes the bolus through the mouth into the opening of the pharynx, and by the coordinated work of the pharyngeal muscles and tongue driving pressure, the bolus reaches the esophagus.^2,3^ During normal swallowing, the movements of each organ are strictly controlled by the neuromuscular system to avoid the accidental transfer of food and liquid into the respiratory tract.^2,4^ When the physiological mechanisms supporting the swallowing sequence are altered, a residue of bolus may invade the pharynx, leading to life-threatening aspiration pneumonia.^4^ Dysphagia, the swallowing difficulty, is a symptom common to a multitude of diseases and can affect each phase of deglutition, resulting in malnutrition and deterioration in the quality of life.^1,2,5^ Therefore, it is important to evaluate the swallowing functionality before problems arise for preventing severe complications.^1^ Deglutition functions are typically evaluated starting from a medical screening based on the following evaluation tests:^6,7^

(i) Repetitive saliva swallowing test (RSST): the patient is asked to swallow saliva as many times as possible for 30 s.^8^
(ii) Modified water swallowing test (MWST): the patient is given 3 mL of water in the oral vestibule and then instructed to swallow, repeating the action for two or three times.^9,10^

However, the results obtained are subjective, since they require investigator’s expertise for correct interpretation and allow the clinician to grade swallowing movements without a precise quantitative analysis. In medical device-based clinical practice, two techniques are the gold standard for the evaluation of swallowing, videofluorography swallowing study (VFSS)^1,11,12^ and fiberoptic endoscopy evaluation of swallowing (FEES),^12,13^ but they have critical drawbacks. VFSS encompasses significant radiation exposure,^14^ while the ingestion of a barium bolus can be dangerous for a patient with high aspiration risk.^11^ FEES involves passing a fiberoptic laryngoscope trans-nasally to visualize the swallowing act, making it an invasive technique with the possibility of affecting the normal swallowing behavior.^13^ Furthermore, in both cases, the analysis requires technical experts and large and expensive equipment, while frequent examinations are not allowed because of its invasiveness.^4^ Among alternatives, a less invasive analysis exploited in clinical practice is the recording of the electromyography (EMG) of the submental suprahyoid muscles.^5,15^ However, even if different parameters related to the deglutition mechanisms are assessed by EMG, they are mainly linked to the individual anatomical peculiarities,^16^ making these tests suitable only if coupled with other techniques.

In the past years, studies demonstrated that the analysis of hyoid bone activity provides fundamental information about the swallowing quality.^1,17^ This stimulated the development of novel devices based on the evaluation of the laryngeal excursion, exploiting different transduction methods. Some approaches involved the use of a sensor of bending, in which the increase in the bending angle is associated with increased measured resistance,^1^ or dual-axis accelerometers for the detection of swallowing impairments by positioning the sensor on the neck in correspondence to the thyroid cartilage.^18^ Although all these techniques can offer interesting information about the deglutition process, they require the use of brittle and rigid traditional electronic components, which cannot be compliant with the skin, and a voltage supply, which is one of the most critical obstacles for the development of long-term monitoring systems.^19^ A more advanced approach takes advantage of piezoelectric sensors and their ability to generate an output signal as an effect of rapid variations of dynamic pressure. Simultaneous recording of the piezoelectric signal together with traditional clinical techniques demonstrated their ability to precisely follow the laryngeal movement during the bolus passage. Different types of piezoelectric sensors were tested using commercial or ad-hoc designed devices. A PZT-based bulk sensor was used to evaluate the larynx movements for finding the best sensor position on the neck.^12^ The device was taped on the throat by a rigid plastic plate, making this solution uncomfortable for the patient as it affects the normal swallowing behavior. A similar approach incorporated an array of piezoelectric sensors in a resin sheet for observing the stages of the deglutition process.^11^ However, the system was not suitable for the clinical practice as it requires a laboratory system for the signal acquisition and an external operator to hold it in place. Other studies proposed the use of commercial flexible piezoelectric sensors based on PVDF (polyvinylidene difluoride), fixed by adhesive tape wrapped around the neck. Even if this device could follow the curved surface of the neck, the low adhesion to the skin caused a relative movement between the device and the larynx. This approach allowed the measurement of the mechanical behavior of the larynx but was unsuitable to support clinicians during their analysis.^15,17^ Indeed, any attempt to design a PVDF-based device that is able to limit delamination from the skin faces technological issues due to PVDF piezoelectricity degradation because of typical packaging temperature. The solutions mentioned above are all affected by several limitations in terms of conformability and easiness of use, limiting their use in real practice. Based on these considerations, a completely non-invasive and unobtrusive technology, measuring the swallowing process without affecting it, is currently not available.

We report on a new flexible piezoelectric sensor that is able to follow the larynx movement through the skin deformation induced by the hyoid bone pressure. This smart patch is able to provide clinically relevant information about the subject’s swallowing ability and to offer support to the doctor in the quantitative diagnosis of deglutition disorders. The device is based on aluminum nitride (AlN) patterned on a Kapton foil substrate. Its small dimensions, thickness (26 μm), and lightness (less than 2 g) make it completely free to warp around the curvilinear surface of the neck. Therefore, the sensor is able to follow the tiny laryngeal region movement without interfering with the normal swallowing behavior as it avoids the use of a rigid structure on the neck. The biocompatibility and nontoxicity of all the used materials reduce the possibility of allergic reactions or irritations on the subject’s skin. Finally, the piezoelectric sensor was connected to an ad-hoc designed charge amplifier and integrated with a signal conditioning circuit. The implemented output module is able to transmit signals to the smartphone, exploiting Bluetooth technology, guaranteeing a fully unobtrusive feature and easiness of use of the sensing system. This work encompasses the fabrication process of the piezoelectric sensor, its electromechanical characterization by a customized built-up measurement test bed, and the analysis of the piezoelectric patch directly on the human skin. Finally, this device provides an exhaustive evaluation of the ability of the system to support doctors in the real-time monitoring of spontaneous swallowing frequency, single swallowing duration, and latency time.

## Experimental Section

### Sensor Fabrication

**Figure 1:**
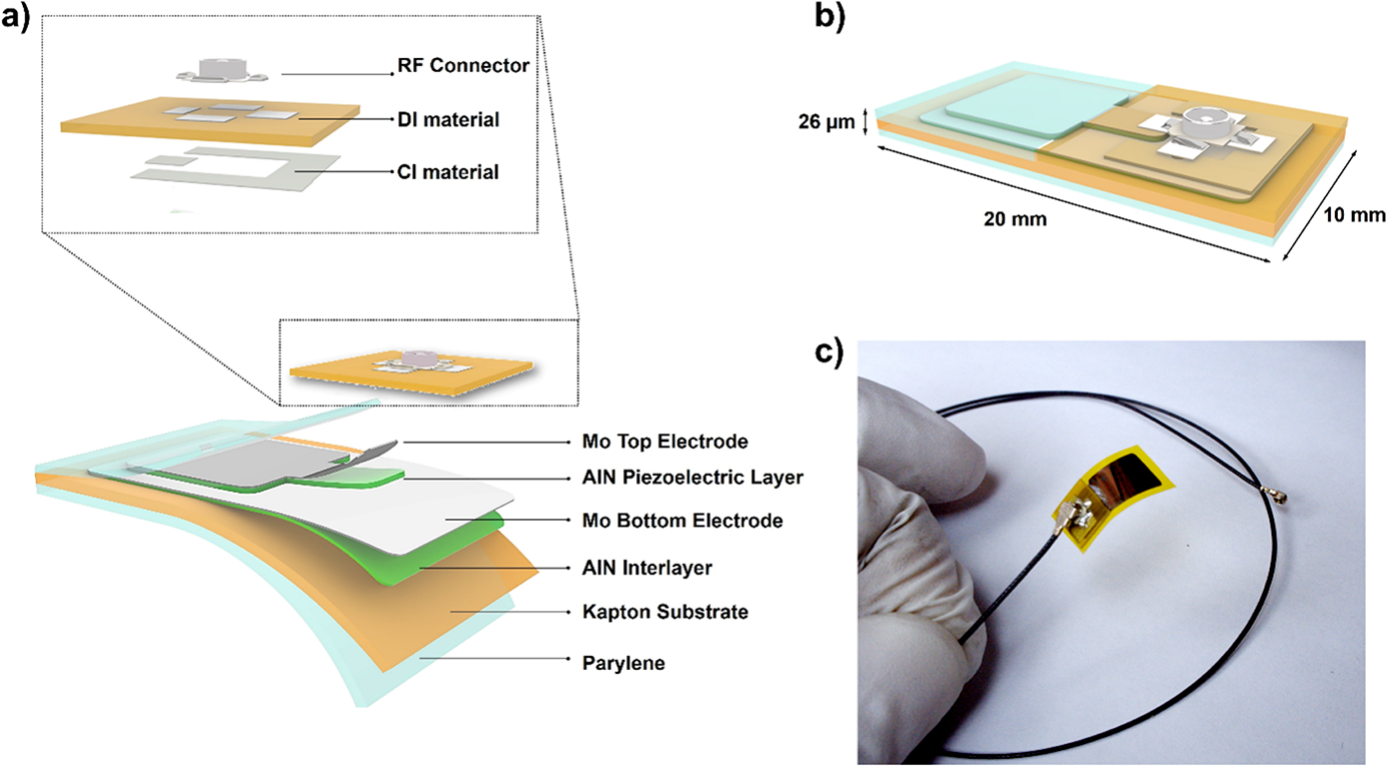
Flexible piezoelectric sensor structure. Exploded schematic drawing of the multilayered flexible transducer (a), with a detailed 3D view of the layers composing the external package (inset). Design and dimensions of the final sensor (b) and a picture of the completely processed sensor (c).

The piezoelectric sensor structure is based on a thin-film heterostructure consisting of an aluminum nitride interlayer (AlN-IL, 120 nm), molybdenum bottom electrode (Mo, 200 nm), piezoelectric aluminum nitride (AlN, 1 μm) layer, and molybdenum top electrode (Mo, 200 nm) stack (Figure 1). The whole fabrication process (reported in the Supporting Information) trades on standard microfabrication techniques including photolithography and sputtering deposition. AlN is considered one of the ideal materials for the development of flexible and compliant piezoelectric sensors for biomedical application. It is intrinsically biocompatible^20^ and can be profitably deposited on polymeric and soft materials, exhibiting its piezoelectric intrinsic property.^21^ It provides high electromechanical coupling^22^ and intrinsically high resistivity,^23^ making it excellent for the electromechanical transduction mechanism. Kapton foil (25 μm thick) was chosen as the main structural layer for the growth of the sensitive layer because of its structural and mechanical properties.^24−26^ An innovative 3D-printing system (DragonFly LDM, Nano Dimension) allowed one to implement metal contact on the electric pads inside the sealed package, embedding the flexible piezoelectric transducer without affecting the device performance. In Figure S2a–f, Supporting Information, we report the printing steps required for the package.

### Laryngeal Motion Simulator (LMS)

**Figure 2:**
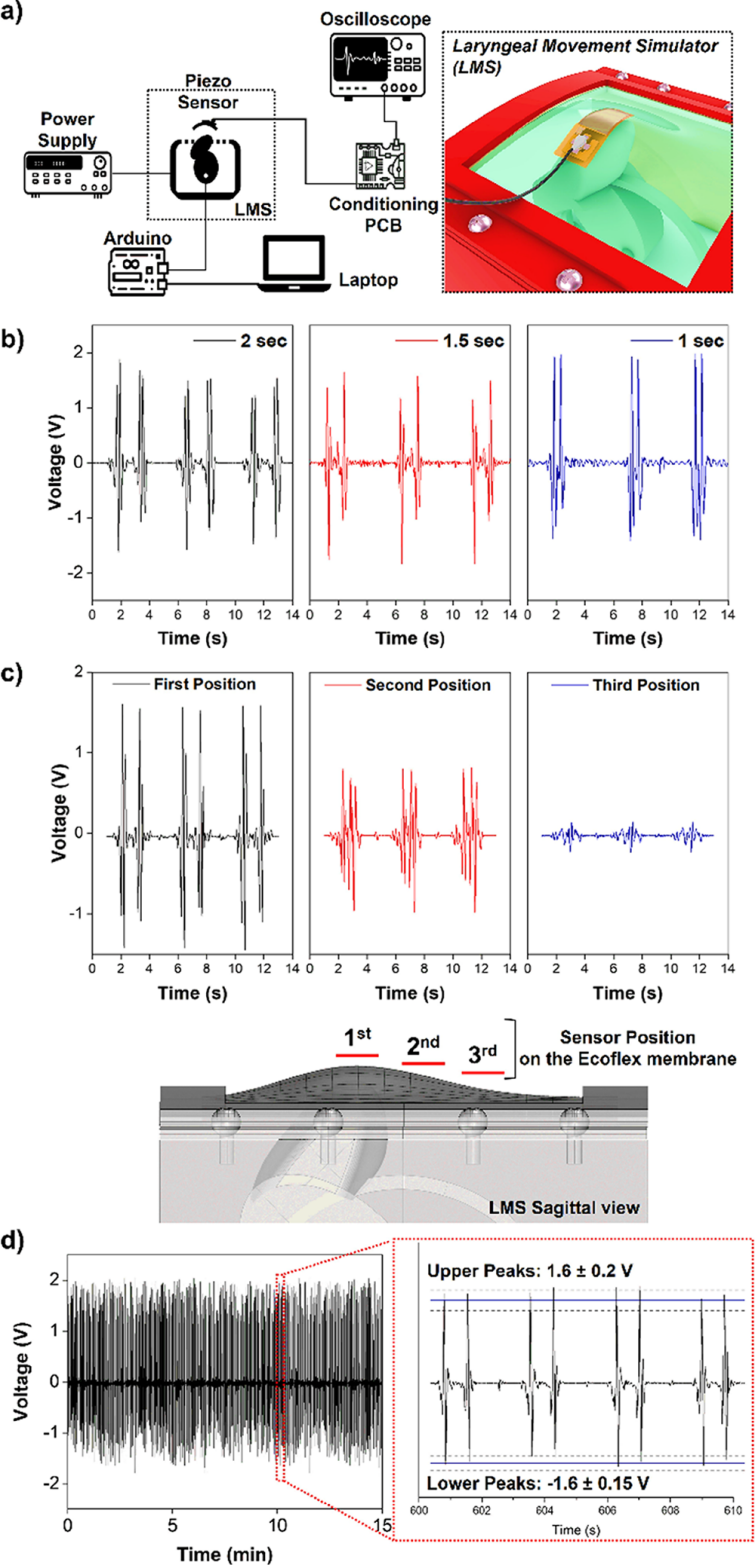
Laryngeal swallowing simulator (LMS). Schematic illustration of the measurement setup for the electromechanical characterization of the piezoelectric sensor (a). Generated output voltage by the sensor at different swallowing periods (b); voltage signal variation due to the position of the sensor respects the initial point of the simulated thyroid cartilage. In the 3D reconstruction, the position of the device is reported (c). Stability test of the sensor output voltage (d). The signal displayed no noticeable fluctuation during the repetitive pushing tests (inset).

A custom electromechanical setup was created to simulate the deglutition, replicating the diagonally upward and downward motions of the laryngeal prominence (Figure 2a). All the parts of this system, hereafter called the laryngeal motion simulator (LMS), were fabricated by a 3D-printer (Creality CR-10S Pro). A stepper motor (Mercury Motor, SM-42BYG011-25) provided the motorized pushing mechanism, whose position and velocity were guided by an Arduino microcontroller unit (Arduino-Nano). The internal ellipsoidal shape of the LMS system was designed to provide the motion to the protruding part, which slides against an Ecoflex (Ecoflex 00-50, Smooth-on) membrane and acts similarly to the thyroid cartilage moving below the neck skin. The LMS was designed for perfectly matching the characteristics of the swallowing act. The Ecoflex flexible membrane offers a suitable test site (about 7 × 7 cm^2^), comparable with the common dimensions of the neck of an adult person, and mimics the mechanical properties of the skin. The protruding part of the LMS ensures a reliable height (about 12 mm) of the simulated laryngeal prominence during its movements against the Ecoflex membrane, in accordance with the data reported in the literature for a normal swallowing.^27^ Finally, the angular velocity value of the stepper motor is set for allowing a complete movement of the protruding part of the LMS in a period compliant with the normal swallowing duration.^28^ The sensor is attached directly on the upper surface of the silicone membrane (Figure 2a, inset). Its output is conditioned before the recording by the use of an ad-hoc designed charge amplifier with an amplification gain of 100 mV/pC, including a low-pass filter with a cut-off frequency of 15 Hz. The sensor output voltage is acquired by an oscilloscope (Tektronix, MDO4000; sampling frequency, 100 samples/s).

### Clinical Protocol and Subjects

The smart patches were tested on the skin of volunteers in their early 30s, with no clinical symptoms or anamnestic history for swallowing disorders. All procedures involving human participants were in accordance with the ethical standards of the institutional research committee and with the 1964 Helsinki Declaration. Processes were approved by the Ethical Committee of Regione Liguria and Ospedale Policlinico San Martino IRCCS (no. 8/2020). No allergic reactions or wounds on the skin were observed in any of our studies, no side effects were recorded, and all the subjects involved in the study feel comfortable with all the procedures.

## Results

In the pharyngeal phase of the deglutition process, the laryngeal prominence traces a path upward and forward and then returns to the original position.^29^ Generally, a piezoelectric transducer positioned at this level produces a typical pattern useful for recording information about the quality of patient swallowing and the analysis of these waveforms provides important clinical information.^4,11^ In particular, a distortion in the deglutition wave profile and a variation of temporal parameters are crucial signs of an undesired variation in the patient deglutition ability.^15,30−32^ The proposed flexible piezoelectric smart patch is extremely light, thin, and flexible enough to compliantly adhere to the neck (Figure 1). The adhesion on the skin is guaranteed by a sticky layer of polydimethylsiloxane–polyethylenimine (PDMS–PEIE) on the backside of the sensor, allowing one to easily position the device like a common patch and to safely remove it after the measurement is completed. This minimizes the mechanical load, avoids physiological constraints during the swallowing process, and impedes the relative movement between the skin and the sensor, improving the signal quality even through long acquisition. According to the literature, the pressure produced by the bolus passage through the pharynx is distributed between 10 and 50 kPa.^33^ In this range, the sensor pressure sensitivity and response time were measured for verifying the sensor’s applicability in the evaluation of the deglutition function. The Supporting Information (Figure S3) describes the characterization procedure. In general, due to the direct piezoelectric effect, the generated output voltage increases with the pressure applied on the sensor. It shows an almost linear relationship within the applied pressure up to 50 kPa with a calculated sensitivity of 0.025 V/N and a response time of 15 ms. These values are suitable for the proposed application and comparable to the recently described pressure sensor.^19,34^

### In Vitro Signal Evaluation by LMS

By the LMS system (Figure 2a), a preliminary set of tests were performed to verify the ability of the sensor to record the swallowing act and to perform a comprehensive characterization of the response as a function of the simulated swallowing process. In an ideal case, the expected output voltage of the transducer, representing the deglutition wave, is referred to two distinct swallowing phases: the upward and downward laryngeal prominence excursions. The first peak of the signal corresponds to the moment when the laryngeal prominence passed and pushed the sensors when moving to the upper position, and the second peak corresponds to the moment when the laryngeal prominence passed and pushed the sensors when returning to the resting position.^11,35^ Different conditions were studied by varying the stepper motor velocity and the mutual position of the sensor with respect to the protruding part of the LMS, according to the following considerations:

(i) A high reproducibility of the signal is a key point for every experimental analysis:^15^ the sensor should measure a variation of the swallowing duration without varying the standard pattern of the deglutition wave.
(ii) The recorded signal shows a higher excursion amplitude and an earlier onset latency if the swallowing is measured at the thyroid cartilage level: the sensor positioning is a crucial aspect to reach a high quality of the acquired signal.^12^
(iii) The chance to analyze the same subject with the same sensor during different experimental procedures is mandatory for limiting medical costs: signal stability can guarantee the repeatability of the measurements.

With respect to point (i), the first characterization test was performed by imposing a variation in the swallowing period. Since the main frequency band of laryngeal motion falls in the 0.5–1 Hz^11,30^ frequency range, the LMS velocity was properly adapted to obtain a complete forward and backward cycle in 1, 1.5, and 2 s. The sensor generated an output voltage close to the expected deglutition wave, composed of the two stages related to the double movements of the simulated thyroid cartilage. As in Figure 2b, the sensor detected this variation properly, displaying the change in the signal duration while preserving the same deglutition waveform. According to point (ii), to determine the best position on the neck, the patch was tested in three different positions with respect of the initial point of the simulated thyroid cartilage (imposed cycle duration, 1.5 s). The first position was defined as the point where the protruding part of the LMS is at the maximum amplitude with respect to the Ecoflex membrane (Figure 2c).

Indeed, this position can be assimilated to the initial location of the thyroid cartilage on the neck. Then, the device was displaced at 15 and 30 mm from this point, defined as second and third positions, respectively, to reflect the common spatial range of the laryngeal movement.^36^Figure 2c shows that the signals recorded at the first position has better signal definition, with higher amplitudes and the expected duration for the imposed cycle time (1.5 s), witnessing higher motion resolution. Nevertheless, signals were still recognizable even in the second and third positions, despite the fact that some features of the deglutition waves were lost because the sensor in those positions is unable to follow the entire path of the protruding part of the system. The reduced signal amplitude and the lower temporal resolution recorded in these two positions can lead to an erroneous assessment of the deglutition function. Therefore, the laryngeal prominence is the most appropriate position to obtain a high-quality signal. To verify the stability of the sensor output during the normal duration of a medical examination, as point (iii), a cyclic measurement of 15 min was performed. Figure 2d shows that the generated output voltage displayed no noticeable fluctuation during the repetitive pushing tests. As a proof of high stability, the value of the amplitude peaks was constant during the whole recording (mean value ± standard deviation) (Figure 2d, inset).

### On-Skin Swallowing Detection

**Figure 3:**
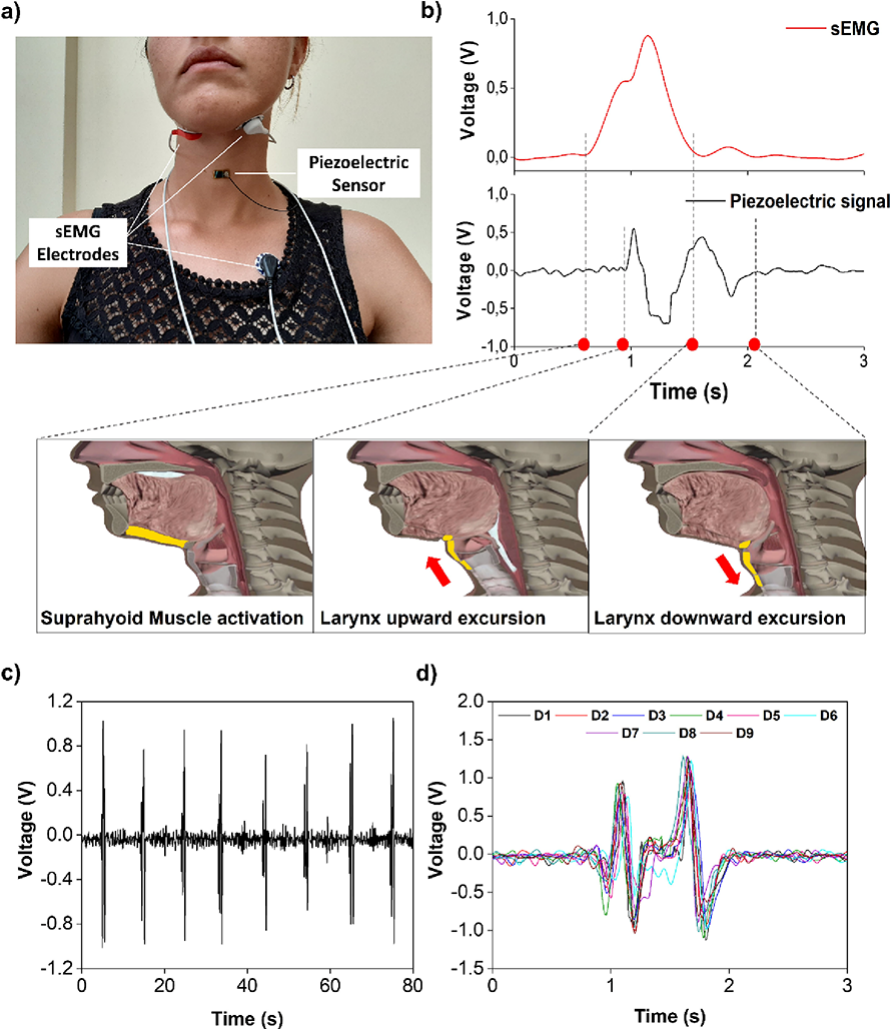
Electromechanical analysis. Deglutition wave recognition and segmentation. Picture of the positioning of the piezoelectric sensor on the neck and of the sEMG electrodes (a). Comparison between the recorded signals of a single swallow action: piezoelectric sensor vs sEMG (b). The sequential correlation between time points on the signal and the deglutition event is shown. An entire experimental procedure is reported (c), in which it is possible to observe the nine consecutive swallows, and then segmented and overlapped (d).

**Figure 4:**
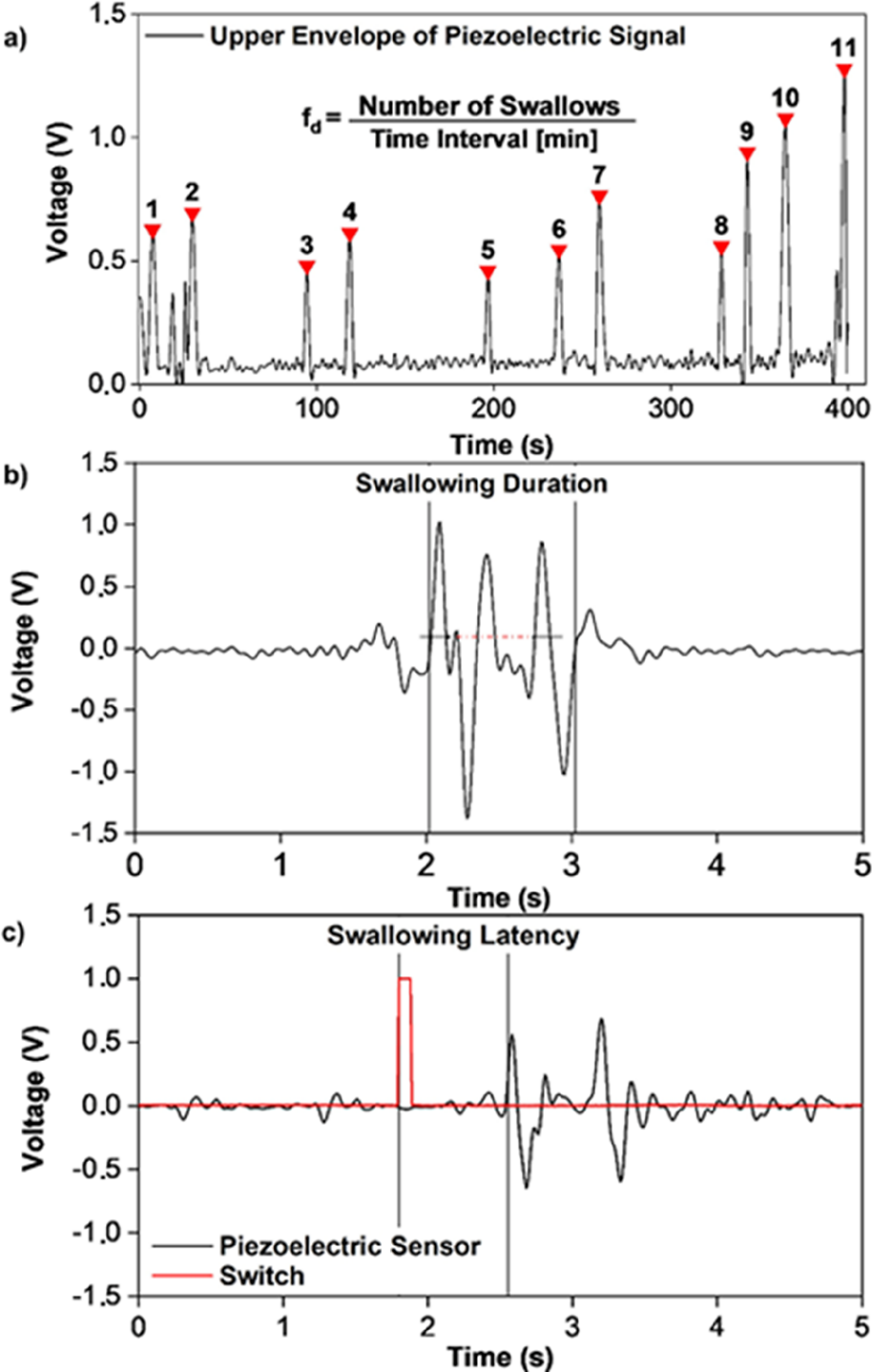
Measurement of *f*_d_, *t*_d_, and *L*_d_. Autonomous swallowing peak identifications in an ideal case using the double threshold method (a); spontaneous swallowing frequency is defined as the number of act per minute. Description of the measurements of swallowing duration (b) and latency (c).

**Figure 5:**
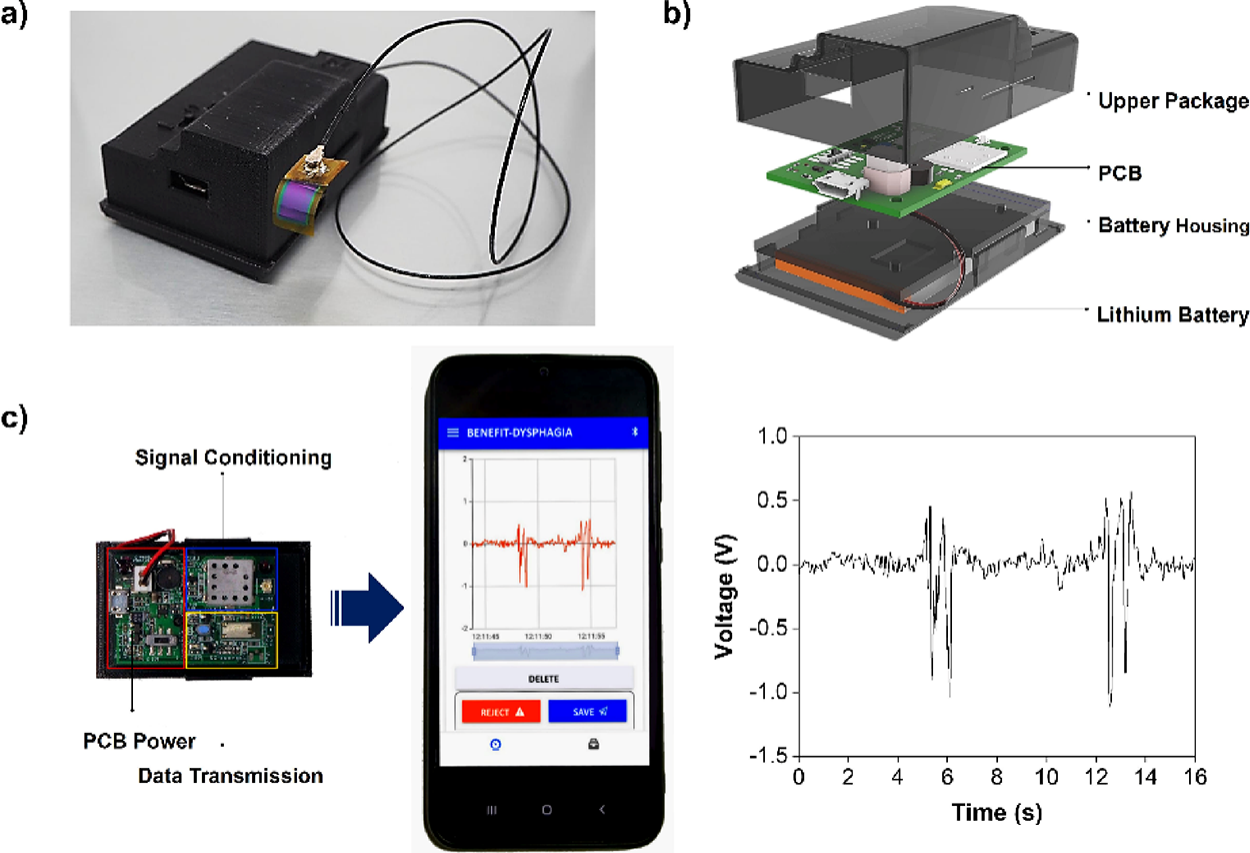
Electronic conditioning system and wireless data transfer. A picture of the complete device is shown in panel (a). In panel (b), an exploded schematic illustration of the system integrated circuit is shown. (c) Wireless transmission of the swallowing signal to a smartphone compared with the same acquisition by the oscilloscope.

A combination of electrophysiological and mechanical analyses was carried out to univocally associate each portion of the piezoelectric signal to the temporal events during deglutition. Figures 3–5 show a series of tests referring to representative subjects only with the purpose of explaining the method; the reported results belong to different volunteers.

The proposed test involved the recording of nine consecutive swallows interspersed for 10 s. The volunteer was trained to hold 10 mL of water in his mouth until he was instructed to swallow by the operator. The sensor was combined to a surface electromyography (sEMG) device^12,30^ (SHIELD-EKG-EMG bio-feedback shield-OLIMEX) to simultaneously record the suprahyoid muscle activity and the thyroid cartilage movement. Three surface electrodes were used: two electrodes were symmetrically applied under the chin with an interelectrode distance of 30 mm, and a reference electrode was attached in the upper part of the chest (Figure 3a). The generated signal was postprocessed with a band-pass filter between 20 and 400 Hz and smoothed using a 10-point adjacent averaging filter.^12^ Rectification and an envelope of the signal were obtained. The sensor was conformally attached directly on the neck in correspondence to the laryngeal prominence. Both the systems were wire-connected to the oscilloscope to avoid any time delay between the recorded electrical signals. The signals acquired by the sensor during the measurement show the repetition of two phases related to the rising and descending movements of the thyroid cartilage as demonstrated by the LMS. The onset of sEMG identifies the start of the oral phase of the deglutition and it bursts the laryngeal upward excursion.^37,38^ The temporal sequence of these biomechanical events was brought to a latency period between the sEMG onset and the first deflection in the signal generated by the sensor, as observed in the graph reported in Figure 3b. The duration of this period, 280 ± 60 ms, was consistent with the results reported in previous studies for healthy people.^12,30^ As soon as the work of the suprahyoid muscles ceased, the tongue force disappears and the thyroid cartilage starts to move back to the neutral position.^39^ In accordance with this observation, the second part of the piezoelectric signal started just after the offset of the sEMG signal, confirming the hypothesis that this phase was related to the laryngeal structures descending. Then, an evaluation of the repeatability of the piezoelectric signal was necessary. The entire output signal (Figure 3c) was segmented in nine different parts, each including a single deglutition waveform; the reproducibility was qualitatively ascertained, overlapping the recorded waveforms and analyzing them by a cross-correlation approach by the extrapolation of Pearson’s correlation coefficient (PCC).^40^ As shown in Figure 3d, the overlapping of the segmented signal emphasizes the high similarity among the recorded piezoelectric signals. The PCC values for the different deglutition (reported in Table S1) ranged between 0.79 and 0.91 with a mean value of 0.88, confirming a significant positive correlation and high reproducibility of the consecutive swallowing acts of the subject.

### Measurement of Clinically Relevant Parameters for Assessing the Swallowing Quality

Considering the high quality of the recorded signal, it was possible to introduce the recognition of some features, which are useful for the evaluation of the swallowing quality. In this respect, the most clinically significant information is generally provided by a temporal analysis of the deglutition pattern. Three parameters were identified:

(i) Spontaneous swallowing frequency (*f*_d_ expressed as the number of swallowing per minute). Evaluation of the frequency of spontaneous saliva deglutition.^41^
(ii) Duration time of a swallowing act (*t*_d_). This parameter has a wide physio-pathological importance since a safe and efficient swallowing is strictly related to a coordinated action of at least 15 pairs of muscles.^41,42^
(iii) Latency (*L*_d_), expressed as the duration of the period from the instruction to swallow to the beginning of the signal related to the larynx movement. A delay of the swallowing reflex is a common presentation in neurogenic dysphagia.^12^

Commonly, clinicians rely on palpation and observation of the thyroid cartilage elevation to estimate this information but without any objective data.^12,43,44^ To test our sensor’s ability to evaluate the parameters described above, we positioned it on the neck of eight different volunteers. The swallowing frequency (Figure 4a) was measured for a period of 400 s while the subjects sat upright on a chair and were instructed to behave naturally but limiting any head movement.

The acquired signals were postprocessed to automatically calculate the frequency value *f*_d_. In this respect, the upper envelope of the signal was obtained to simulate a digital logic, in which the signal is different from zero only when a swallowing is present. The average windows chosen for the envelope allowed one to keep all the signal information and to extrapolate the single swallows. Then, the peaks were counted by a double threshold system, in which the first one defined the amplitude level for the peak detection and the second one avoided counting two consecutive peaks, considering a minimum pause of 2 s between two consecutive spontaneous swallows. Figure 4a shows that this approach clearly distinguished the autonomous swallowing from the noisy base line. For all the subjects under testing, the mean *f*_d_ values, as reported in Table 1, were between 1.5 ± 0.2 and 4.1 ± 0.6 swallows/min, respectively, during about 6 min of recording, comparable with the results reported in the literature for normal subjects.^43^ To calculate the mean *t*_d_, the experimental procedure followed the rules described for the evaluation of the repeatability of the signal. The swallowing duration was intended as the period between the onset of the first deflection and the return to the baseline of the last one (Figure 4b). For each subject, all the swallows were considered in the analysis and the extrapolated values were then averaged and expressed as mean ± standard deviation. The results are reported in Table 1 with mean values ranging from *t*_d_ of 0.9 ± 0.1 to 1.7 ± 0.5 s, in accordance with data recorded on normal subjects.^45^ Finally, to evaluate the latency *L*_d_, the swallowing waveform was recorded alongside a trigger signal that was released simultaneously to the swallowing order by the operator. The *L*_d_ was calculated as the distance between the rising edge of the trigger signal and the onset of the first deflection of the swallowing signal (Figure 4c). The results are summarized in Table 1, and the range of the obtained values (mean ± std) is in good agreement with the literature.^11^

**Table 1:** On-Skin Analysis Results^a^

	spontaneous swallowing frequency *f*_d_ (swallows/min)	swallowing duration *t*_d_ (s)	latency *L*_d_ (ms)
S1	1.6 ± 0.1	1.11 ± 0.07	310 ± 70
S2	2.2 ± 0.2	1.42 ± 0.5	360 ± 60
S3	2.13 ± 0.05	1.2 ± 0.4	420 ± 90
S4	2.4 ± 0.3	1.7 ± 0.5	800 ± 90
S5	1.9 ± 0.3	1.2 ± 0.3	760 ± 50
S6	4.1 ± 0.6	1.6 ± 0.3	700 ± 100
S7	3.5 ± 0.5	1.13 ± 0.09	560 ± 30
S8	1.5 ± 0.2	0.9 ± 0.1	630 ± 90

a Summary of the quantitative analysis of spontaneous swallowing frequency, swallowing duration, and latency for the different subjects S1–S8. All the values are reported as mean ± std.

However, to validate the applicability of this technology to the point of care, the original conditioning circuitry was improved by adding a Li-poly battery and a wireless data transmission system (NRF51822-QFAA Nordic Semiconductor), composed of an ultralow-power system-on-chip (SoC) ideally suited for Bluetooth Low Energy (about 50 mW of energy consumption with one transmission channel and 100 Hz of sampling frequency), which is able to transfer the recorded signal generated by the sensor (Figure 5a,b).The battery supply guarantees up to 3 h of autonomy. Figure 5c shows a picture of the signal conditioning/transmission unit connected with an Android-based smartphone that displays the swallowing signal by the use on an ad-hoc developed app (Video S1 in the Supporting Information). The received output presented a similar wave pattern compared to the signal obtained from the measurement system registered by the oscilloscope.

## Conclusions

The presented results reported the successful use of an ultrathin and flexible piezoelectric sensor for the evaluation of the swallowing quality and its possible application as a medical device to provide clinically relevant information in a non-invasive way. The ultralight sensor based on AlN was fabricated and patterned by traditional microfabrication techniques, on a thin layer of Kapton, and coupled with the skin by a layer of sticky PDMS–PEIE polymer. When coupled with the neck of the subject, its conformable structure, together with the sensitive and predictable response, offers high performances without affecting the normal behavior of the larynx movement. The output voltage was amplified and filtered by a proper conditioning system and successfully transferred to a smartphone by exploiting wireless Bluetooth technology, allowing its seamless use in therapeutic applications. The sensor was able to continuously provide a repeatable output even during long experimental procedures. The physician, exploiting the generated data, could measure some important factors, such as the duration of the swallowing act, frequency of spontaneous saliva deglutition, and latency. The recording of these features permits the objective evaluation of the subject’s swallowing ability and could provide an early diagnosis of pathological conditions. In this respect, we are currently investigating the possibility to create a system that is able to provide an automatic and real-time extrapolation of clinically relevant information and to test it in a pivotal clinical study to establish its safety and effectiveness. In conclusion, it will be possible to push the limits of current diagnosis systems in the swallowing analysis, suggesting an innovative technological approach to the clinical evaluation of the patients’ health conditions.

## Supporting Information Available

Materials and Methods: sensor fabrication, packaging and electrical connections of the sensors, and on-skin adhesion; Figure S1: schematic 3D representation of the main microfabrication process steps for the production of sensors; Figure S2: schematic 3D representation of the main printing steps required for packaging and electrical connection of the sensors; sensor characterization; Figure S3: sensor properties; Table S1: Pearson’s correlation coefficients (PDF)
Video of a swallowing test (MP4)
