# Supplementary material for: Conformable AlN Piezoelectric Sensors as a Non-invasive Approach for Swallowing Disorder Assessment
Source: ACS Sens. 2021 May 19;6(5):1761–9. doi: 10.1021/acssensors.0c02339 (PMC8294609; doi:10.1021/acssensors.0c02339)
Supplement: Supplementary file 1 — se0c02339_si_001.pdf [file se0c02339_si_001.pdf]

Conformable AlN piezoelectric sensors as non-invasive approach for swallowing disorders assessment

Lara Natta*, Francesco Guido*, Luciana Algieri, Vincenzo M. Mastronardi, Francesco Rizzi, Elisa Scarpa, Antonio Qualtieri, Maria T. Todaro, Vincenzo Sallustio and Massimo De Vittorio.

E-mail: lara.natta@iit.it, francesco.guido@iit.it

L. Natta, F. Guido, L. Algieri, V. M. Mastronardi, F. Rizzi, E. Scarpa, A. Qualtieri, M. De Vittorio
Istituto Italiano di Tecnologia, Center for Biomolecular Nanotechnologies, 73010, Arnesano, Le, Italy
E-mail: lara.natta@iit.it, francesco.guido@iit.it

F. Guido, L. Algieri
Piezoskin S.r.l. 73100, Lecce, Italy

M. T. Todaro
Istituto di Nanotecnologia, Consiglio Nazionale delle Ricerche, c/o Campus Ecotekne, Via Monteroni - 73100 Lecce

V. Sallustio
Hospital Unit Phoniatrics and Communication Disorders, Rehabilitation Department, ASL Lecce, 73100, Lecce, Italy

M. De Vittorio
Università del Salento, 73100, Lecce, Italy

Supporting Information

MATERIALS AND METHOD: SENSOR FABRICATION

The microfabrication process (S1 a-f) starts with the lamination of the substrate (Kapton foil) on silicon (Si) wafer using a thin PDMS sticking layer. The heterostructure was sputtered onto Kapton foil and patterned by optical lithography and chemical etching. In particular, the AlN interlayer (120 nm) was deposited using a pure Al target (99.9995%) in a mixture of Ar and N2 gases (ratio 1:1), applying direct current (DC) pulsed power density (4.11 W/cm2, with frequency of 100 KHz and pulse duration of 1 μs) at working pressure of 2.8×10^{-3} mbar. The AlN interlayer provided for a template to reduce the amorphous polyimide surface and promote higher columnar orientation of the grown piezoelectric AlN film. Moreover, this strategy guarantees the best adhesion of the whole heterostructure on the polymeric substrate1. The Mo bottom electrode (200 nm) was deposited in the same run using a pure Mo target (99.95%) in pure Ar atmosphere under DC power density (2.46 W/cm^2) at working pressure of 5.0×10^{-3} mbar. The patterning of Mo bottom electrode and AlN interlayer was performed by dry etching with ICP-RIE (Inductively Coupled Plasma - Reactive Ion Etching) system using two different gas mixtures, respectively BCl3 and N2 (ratio 9:4) for Mo layer and BCl3 and Ar (ratio 4:1), applying a power to the plate and to the coil of 250 W and 600 W, in both cases. The AlN piezoelectric film and top Mo electrode were deposited in the same run. The piezoelectric AlN film was sputtered without heating the substrate, using a pure Al target (99.9995%) in a mixture of Ar and N2 gases (ratio 1:1) under DC pulsed power density (5.48 W/cm^2, frequency of 100 KHz and pulse duration of 1 μs) at a working pressure of 2.8×10^{-3} mbar. The Mo top layer was sputtered exploiting the same conditions of the bottom electrode. These two films were finally patterned by ICP-RIE etching system under the same conditions of the previous etching step. After the patterning step, the multilayered stack was uniformly coated with a 1 μm-thick Parylene film deposited by chemical vapor coating (Specialty Coating System PDS 2010 Labcoater), for electrical insulation and waterproofing. Vias for electrical connections were then opened through the Parylene layer by oxygen plasma etching. The device was finally cut and peeled off from the rigid Si substrate. The final device had a thickness of about 26 μm and a total area of 2 cm^2.

MATERIALS AND METHOD: PACKAGING AND ELECTRICAL CONNECTIONS OF THE SENSORS

The metal contact is one of the most crucial parts in ohmic-contact MEMS devices and particularly for soft and flexible devices where the electrical connectors are typically difficult to produce. The chance to produce electric pads in a sealed package, which embeds the flexible piezoelectric transducer, is an innovative fabrication aspect of the sensor presented in this work.

Figure S2 reports the printing steps required for the package fabrication by using the 3D Printer (DragonFly LD, Nano Dimensions). The innovative 3D printing system is fitted with two print heads, one for nano Silver conductive ink (AgCite, Ag nanoparticles based) and the other for dielectric polymer ink (DI1086A), able to deposit the materials in the same process.

The 3D printing system allows building a 3D layer-by-layer structure directly on Kapton substrate (a). The printheads are equipped with hundreds of nozzles able to jetting, with extreme precision, both conductive (CI) and dielectric (DI) inks, with a proper designed shape according to the electrodes profile (b). The package fabrication proceeds with a further 3D layer-by-layer structure of CI and DI to shape the electrodes vias and the platform for radio frequency (RF) connector (c) (d). The selection of Hirose U.FL as RF connector represents an important design choice in sensor presented in this work. Indeed, it is the smallest available RF connector and allows maintaining electrical performance without losing lightness and flexibility. The RF connector is finally easily welds on sensor (e-f).

MATERIALS AND METHOD: ON SKIN ADHESION

The conformal attachment of the piezoelectric sensor onto human skin should respect some specific features: the contact between the flexible device and the skin needs to be strong enough to allow a correct measurement; the process requires an ease detachment after use to avoid epidermal reactions; a very low mechanical interfacial stress is necessary to avoid any constrain for the subject. For these reasons, a further thin layer was added on the back of the sensor (the side which is in contact with the human skin). A soft, stretchable and sticky polydimethylsiloxane (PDMS)-based elastomer was obtained by adding a small quantity of a solution of ethoxylated polyethylenimine (PEIE), an amine-based polymer, into the solution of silicon base and curing agent[2]. This additive material is able to significantly reduce the PDMS Elastic modulus (from 1MPa up to 24 kPa) making it more compliant with the skin, and to increase the development of van der Waals interactions with the substrate due to the PDMS-PEIE viscous surface adaptation[2].

FIGURE S1: SCHEMATIC 3D REPRESENTATION OF THE MAIN MICROFABRICATION PROCESS STEPS FOR THE PRODUCTION OF SENSORS.

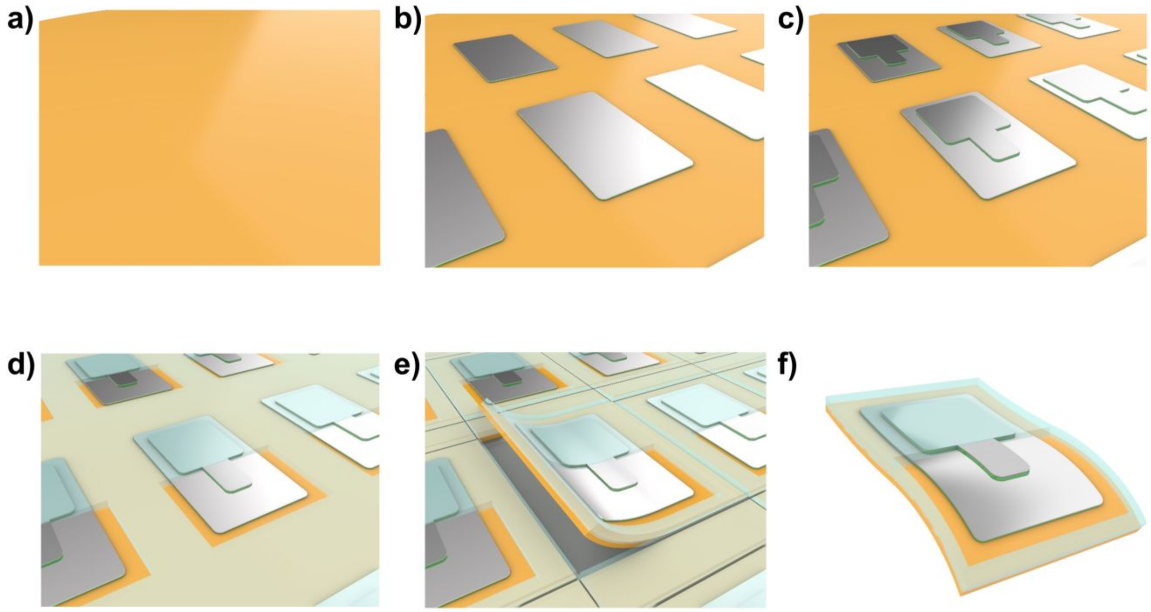

Figure S1: A sacrificial PDMS layer is deposited on the Silicon wafer and the Kapton foil is laminated on it (a). A thin film stack composed of AlN interlayer (120 nm) and Mo bottom electrode (200 nm) is deposited and patterned by photolithography and dry etching (b). Deposition and modeling is made also to piezoelectric AlN (1 µm) and Mo top electrode (200 nm) (c). An insulating 1 µm-thick film of Parylene is deposited on the all surface the sensor. Vias for electrical connection are opened by oxygen plasma (d). The sensors are cut and released (e). Sensor is ready for the electrical connections (f).

FIGURE S2: SCHEMATIC 3D REPRESENTATION OF THE MAIN PRINTING STEPS REQUIRED FOR PACKAGE AND ELECTRICAL CONNECTION OF THE SENSORS.

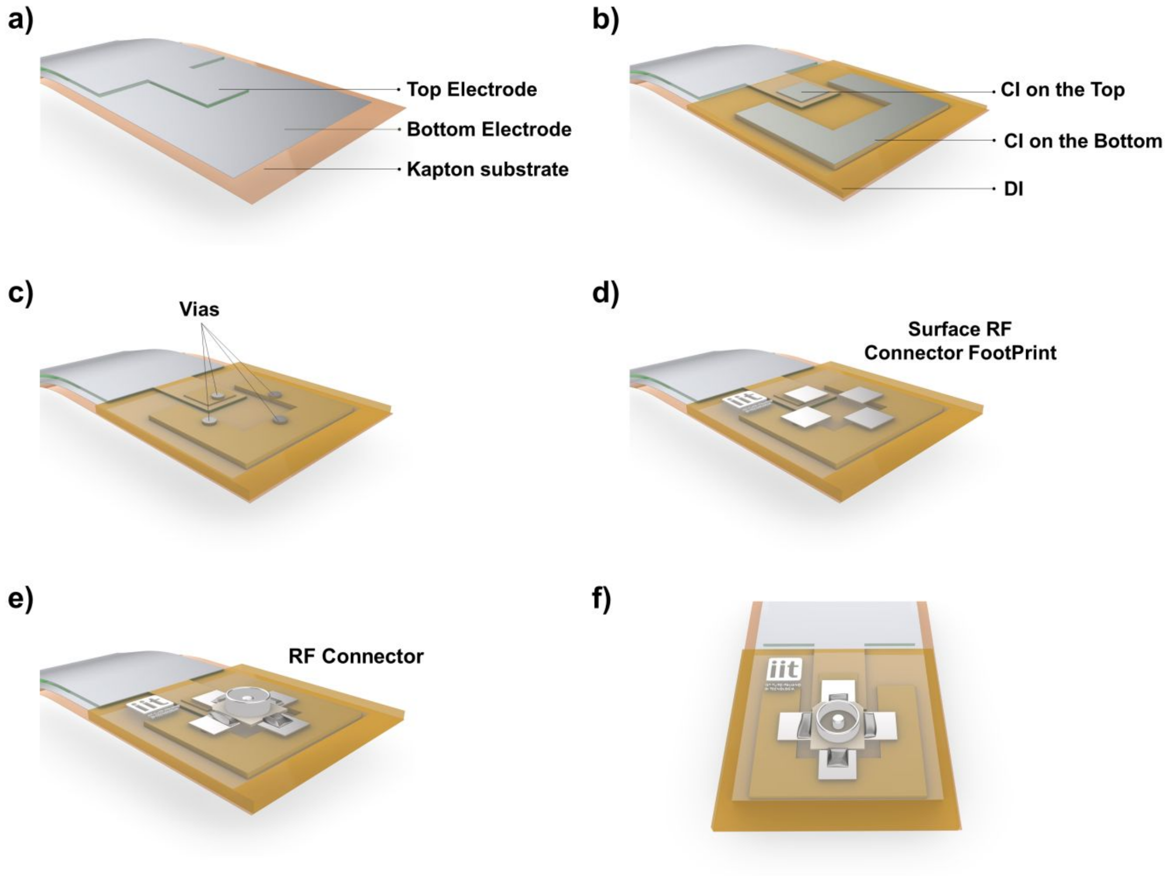

Figure S2: Schematic illustration of the sensor placed in the 3D printer (a). A concurrent deposition of CI and DI inks is made on bottom and top electrode (b). Vias are deposited in order to connect the sensor electrical pads with the RF connection pads (c). Platforms for RF connection are printed (d). RF connector is weld on sensor (e). Sensor is ready for testing (*f*).

SENSOR CHARACTERIZATION

In order to characterized the developed sensor and to evaluate its ability to identify the swallowing biosignal arising from the pharyngeal phase pressure range (between 10 kPa and 50 kPa[3]) and to convert it into an electrical signal, an ad-hoc measurement set up was developed. To generate on the transducer a controlled pressure value, the sensor was attached on a PDMS membrane (500μm- thick) mounted on a small chamber. A pulsed air flux was inflated at controlled pressure, by the use of a solenoid valve controlled by Arduino. The pressure was manually controlled by the use of a manometer. The measurement set-up was calibrated, in terms of applied pressure on the membrane in the pressure range of the swallowing pressure (SP), by using a pull shear instrument (XYZTech Sigma Condor), that can detect the force applied on a calibrated tool with a known surface. In this case a plane tool was used with a circular surface with a radius of 3.5 mm. In Fig. S3a the calibration curve of the measurement set up has been reported. The measurement set up was then used to induce a deformation on the piezoelectric sensor in the desired pressure range between 10 kPa up to 50 kPa with a step of 10 kPa. The generated open circuit voltage was measured by an oscilloscope, without any amplification or filter for the signal conditioning. The obtained results, reported in Fig. S3b, show that the piezoelectric transducers generate a voltage that linearly increases with the applied pressure on the PDMS supporting membrane with a calculated sensitivity of 0,025 V/N. This dependence can be written as in the further equation S1 [4].

$$V_{piezo} = \alpha A_{contact} P \qquad \text{Eq. S1}$$

where P is the applied pressure, $A_{contact}$ is the contact area between the smart patch and the PDMS pushing membrane and α is a parameter that depends on the materials constants for the stack and its deformation mechanics.

Then in order to evaluate the response time of the system a constant pressure of 40 kPa was applied on the membrane in a periodic mode. The corresponding generated voltage was then acquired. As shown in Fig. S3c, the system, exhibits a response time (calculates as the rise time between the 10% and 90% of the signal ascending edge of 15 ms.

FIGURE S3: SENSOR CHARACTERIZATION

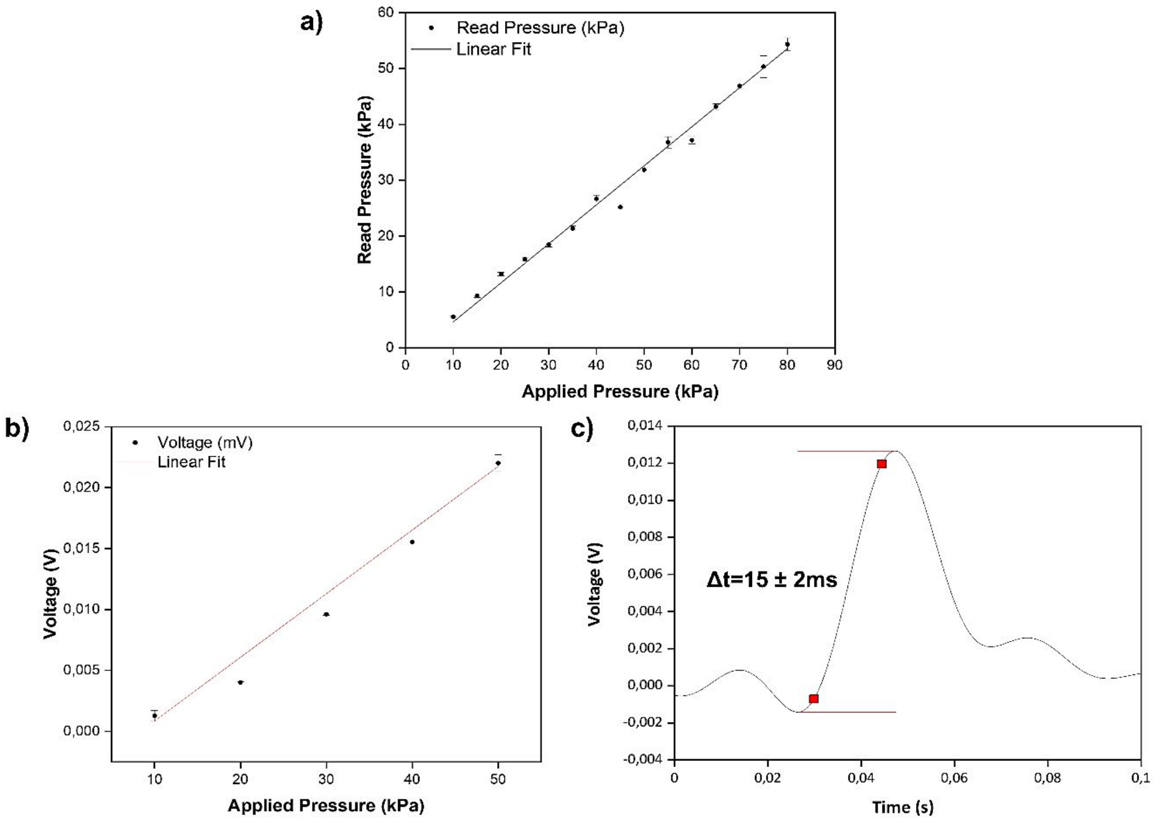

Figure S3: (a) Calibration curve for the measurement setup. The graph that relates the manometer applied pressure, and the pressure measured by the pull shear. (b) piezoelectric sensor response to a pressure variation between 10kPa and 50kPa, the calculated sensitivity is of 0.025 V/N. (c) Sensor response time to the application of an impulsive normal force of 40kPa.

TABLE S1: PEARSON'S CORRELATION COEFFICIENTS

	D1	D2	D3	D4	D5	D6	D7	D8	D9
**D1**	1								
**D2**	0.90	1							
**D3**	0.87	0.89	1						
**D4**	0.88	0.85	0.86	1					
**D5**	0.91	0.85	0.89	0.88	1				
**D6**	0.81	0.84	0.79	0.80	0.80	1			
**D7**	0.85	0.86	0.87	0.89	0.87	0.85	1		
**D8**	0.88	0.85	0.86	0.87	0.89	0.80	0.87	1	
**D9**	0.83	0.83	0.87	0.89	0.90	0.82	0.90	0.90	1

Legend:
1 Perfect Correlation
0 No Correlation

Table S1. Statistical analysis by a cross-correlation approach to extrapolate Pearson's correlation coefficients (PCC) between the nine swallowing acts (D1-D9).

MOVIE S1: VIDEO OF A SWALLOWING TEST

In this video is reported a complete measurement process. The sensor is applied on the subject neck in correspondence of the laryngeal prominence and connected to the measurement conditioning system. The test involved the deglutition of three small glass of water (about 10 mL) and the recorded signal is wirelessly send to a smartphone. The signal is directly visible on the phone monitor just after the test finished.

REFERENCES

1. Kamohara, T.; Akiyama, M.; Ueno, N.; Kuwano, N., Improvement in crystal orientation of AlN thin films prepared on Mo electrodes using AlN interlayers. Ceramics international 2008, 34 (4), 985-989.
2. Jeong, S. H.; Zhang, S.; Hjort, K.; Hilborn, J.; Wu, Z., PDMS-based elastomer tuned soft, stretchable, and sticky for epidermal electronics. Advanced Materials 2016, 28 (28), 5830-5836.
3. Matsubara, K.; Kumai, Y.; Samejima, Y.; Yumoto, E., Swallowing pressure and pressure profiles in young healthy adults. The Laryngoscope 2014, 124 (3), 711-717.
4. Dagdeviren, C.; Su, Y.; Joe, P.; Yona, R.; Liu, Y.; Kim, Y.-S.; Huang, Y.; Damadoran, A. R.; Xia, J.; Martin, L. W., Conformable amplified lead zirconate titanate sensors with enhanced piezoelectric response for cutaneous pressure monitoring. Nature communications 2014, 5 (1), 1-10.
